# Papers and Reports from Special Committees

**Published:** 1865-07

**Authors:** 


Papers and reports from special committees appointed or continued at the last session, being next in order, were called for, and the following were presented:—

On Insanity—Report by Dr. H. R. Storer, of Boston. Referred to the Section on Practical Medicine and Obstetrics.

On the Relations of Electricity to the Causes of Disease—Paper from Dr. Littell, of Pennsylvania. Referred to the Section on Practical Medicine and Obstetrics.

On Climatology and Epidemic Diseases of California—Reports by Drs. Logan, of California, and B. H. Catlin, of Connecticut. Referred to the Section on Meteorology and Epidemics.

On Alcohol and its relations to Man—Report by Dr. G. E. Morgan, of New York. Referred to the Section on Practical Medicine and Obstetrics.

On Autopsies and their Relations to Medical Jurisprudence—Report by Dr. T. C. Finnell, of New York. Referred to the Section on Medical Jurisprudence.

On the Introduction of Disease by Commerce, and the Means of its Prevention—Report by Dr. A. N. Bell, of Brooklyn, N.Y. Referred to the Section embracing Hygiene.

On Exsections and their Relation to Conservative Surgery—Papers by Dr. Tewksbury, of Iowa, and Dr. Lyon. Referred to the Section on Surgery.

On Specialists and Specialties—The Committee were requested to report on Wednesday morning at 9 o'clock.

On the Rank of Medical Corps in the Army—Report by Dr. Tripler, U.S.A. Referred to the general session of Wednesday.

On the Rank of Medical Corps in the Navy—Report by Drs. Anderson, of New York, and others. Referred to the general session of Wednesday.

On Small-Pox—Papers by Drs. Ramsey, of New York, and Nebinger, of Philadelphia. Referred to the Section on Hygiene.

